# Hyposialylation Must Be Considered to Develop Future Therapies in Autoimmune Diseases

**DOI:** 10.3390/ijms22073402

**Published:** 2021-03-26

**Authors:** Anne Bordron, Marie Morel, Cristina Bagacean, Maryvonne Dueymes, Pierre Pochard, Anne Harduin-Lepers, Christophe Jamin, Jacques-Olivier Pers

**Affiliations:** 1 Univ Brest, Inserm, LBAI, UMR1227 Brest, France; marie.morel@univ-brest.fr (M.M.); Cristina.Bagacean@univ-brest.fr (C.B.); maryvonne.dueymes@chu-brest.fr (M.D.); christophe.jamin@univ-brest.fr (C.J.); pers@univ-brest.fr (J.-O.P.); 2 CHU de Brest, Laboratory of Immunolgy, 29200 Brest, France; pierre.pochard@chu-brest.fr; 3 Univ. Lille, CNRS UMR 8576—UGSF—Unité de Glycobiologie Structurale et Fonctionnelle, 59000 Lille, France; anne.harduin-lepers@univ-lille.fr

**Keywords:** sialic acid, sialyltransferase, autoimmune diseases, immunoglobulin, CD22, therapies

## Abstract

Autoimmune disease development depends on multiple factors, including genetic and environmental. Abnormalities such as sialylation levels and/or quality have been recently highlighted. The adjunction of sialic acid at the terminal end of glycoproteins and glycolipids is essential for distinguishing between self and non-self-antigens and the control of pro- or anti-inflammatory immune reactions. In autoimmunity, hyposialylation is responsible for chronic inflammation, the anarchic activation of the immune system and organ lesions. A detailed characterization of this mechanism is a key element for improving the understanding of these diseases and the development of innovative therapies. This review focuses on the impact of sialylation in autoimmunity in order to determine future treatments based on the regulation of hyposialylation.

## 1. Introduction

Over 80 different autoimmune diseases have been reported, mainly impacting women (at a ratio of eight women to two men) [1]. The etiology of these diseases is complex, and several are more common in certain ethnic groups. As an example, systemic lupus erythematosus (SLE) affects nearly three times as many African-American or Hispanic individuals than Caucasians. Genetics has been identified as a risk factor in certain autoimmune diseases, such as multiple sclerosis and SLE. In fact, it has been discovered that those diseases have been diagnosed in several members of the same families and precisely diagnosed in homologous twins [2]. Environmental factors—including infectious agents, pollution and human behavior, for example, the use of drugs—favor those diseases, as well [3]. On this matter, procainamide and hydralazine drugs can be used to modify deoxyribonucleic acid (DNA) methylation, leading to the expansion of autoreactive cluster of differentiation (CD)4+ T cells [4]. Regarding the infectious agent impact, several mechanisms have been described, suggesting a molecular mimicry between the infection agent and self-molecules, leading to the development of autoreactive T cells [5]. Furthermore, epidemiological studies have suggested that smoking is an independent risk factor for several autoimmune diseases such as SLE, rheumatoid arthritis (RA) and systemic sclerosis [6]. Finally, age is suspected to be important, for example, in female patients with RA due to a decrease in estrogen production. This hormone influences the induction of the β-galactoside α2,6-sialyltransferases 1 (ST6Gal I) in plasmablasts. In these cells, the role of this enzyme is to add sialic acid (Sia) residues, notably on the Fc (Fragment, crystallizable) of type G immunoglobulins (IgG). Consequently, in a menopausal condition, the rate of IgG (and particularly IgG autoantibodies with a low Sia content on their Fc) increases and contributes to the development of a proinflammatory environment [7]. The Fc region is responsible for the implementation of physiological effects, including cell lysis, the degranulation of mast cells, basophils, eosinophils and opsonization [8]. The regulation of these effects is induced by variations of Fc glycosylation, such as sialylation and galactosylation as well with, for example, affinity changing of antibodies to Fc receptors on immune cells, or ligation of the first component of classical complement activation [9,10,11].

Recently, glycosylation was highlighted as a factor involved in the development of autoimmune diseases [12]. Glycosylation involves several enzymatic reactions occurring mainly in the endoplasmic reticulum and in the Golgi apparatus, leading to the formation of sophisticated glycans on lipids and proteins. This phenomenon affects cell interactions and the activities of the secreted proteins [13,14,15,16,17]. For example, the glycosylation of T helper (Th) cells modulates their sensitivity to a glycan-binding anti-inflammatory protein: galectin 1. The lack of sialylation on Th1 and Th17 cells makes them vulnerable to galectin-1 mediated cell death, whereas Th2 remains unaffected. Mice that are deficient in galectin-1 develop autoimmune inflammation [18]. A confirmation of this effect has been observed in autoimmune encephalomyelitis models. Galectin-1 binds to ganglioside M1 (GM1) in effector T cells and consequently, inhibits autoimmune manifestations [19]. Another galectin, galectin-9, attenuates Th1 responses and provides protection against the onset of autoimmune diabetes in vivo [20]. Moreover, sialylation modulates cellular functions by other lectins, such as sialic acid-binding immunoglobulin-like lectins (Siglecs), which are immune-modulatory receptors within the mammalian immune system. Among them, CD22 (Siglec-2) appears to be an important negative regulator of B-cell activity [21]. Finally, sialyl motifs, such as sialyl-Lewis X, modulate leukocyte trafficking to secondary lymphoid organs and inflammatory sites through selectins [22].

These observations highlight the important role of sialylation in autoimmune diseases and suggest that targeting sialylation would result in an interesting treatment strategy [23]. The aim of this review is to summarize the impact of sialylation on autoimmune diseases and propose novel therapeutic approaches based on its regulation.

## 2. The Hyposialylation of Autoantibodies and the Generation of Anti-Sialic Acid Antibodies Contribute to the Development of Autoimmune Diseases

### 2.1. The Hyposialylation of Autoantibodies Is Positively Correlated with the Severity of the Disease

Chronic inflammatory demyelinating polyneuropathy (CIDP) is associated with acute inflammatory demyelinating polyradiculoneuropathy and is the most common form of Guillain-Barré syndrome, an inflammatory autoimmune disease of the peripheral nervous system. The level of IgG-Fc sialylation is inversely correlated with CIDP clinical severity [24].

In granulomatosis with polyangiitis—a small-sized vessel-necrotizing vasculitis associated with giant cell granulomas and necrosis—the hyposialylation of IgG is responsible for the modulation of the pathogenic effects of anti-proteinase 3 (anti-PR3) autoantibodies. The hyposialylation of anti-PR3 antibodies is correlated with the severity of the disease [25].

RA is an autoimmune disease associated with strong inflammation and the development of pain in the joints. It is noticeable that, during pregnancy, women have a decrease in RA activity, which is directly correlated to the hypersialylation of IgG Fc, which is linked to estrogen and the stimulation of ST6Gal I in plasmablasts [26]. Confirming this, IgG-Fc sialylation has been shown to be higher in premenopausal women than in men but decreases with age, which is correlated with the emergence of RA in women [27].

### 2.2. The Hyposialylation of Autoantibodies Activates Inflammatory Cells

In granulomatosis, desialylated autoantibodies strongly activate neutrophils and macrophages through Fc gamma receptors (FcγRs). The production of reactive oxygen species and proinflammatory cytokines, such as TNF (tumor necrosis factor), is then induced, strengthening the role of sialylation in the pathogenesis [28]. Interestingly, TNF production is correlated with disease activity [29]. In myeloid cells, TNF has been shown to be able to activate Neu1 sialidase activity, which could explain the hyposialylation of anti-PR3 autoantibodies [30].

In RA, anti-citrullinated protein antibodies (ACPAs) lack of Sia residues on their Fc fragments leads to an increased affinity for FcγRs. The activation of certain inflammatory cells is enhanced as well as the recruitment of complement molecules, thus contributing to chronic inflammation [31,32]. In contrast, the effect of ACPAs can be eliminated by adding Sia residues to the sugar moiety. These results can be observed in mice treated with the Sia precursor, *N*-acetylmannosamine (ManNAc), leading to an increased sialylation of IgG [33]. An explanation of autoantibody hyposialylation lies in the increase of follicular helper T (Tfh) cells and, particularly, interleukin-17-producing Tfh (Tfh17) cells. These cells negatively regulate ST6Gal I from autoantibody-producing B lymphocytes via the OX40-OX40L (TNF receptor superfamily) interaction. The blocking of OX40 prevents the development of RA in K/BxN mice (T cell receptor [TCR] and the MHC class II molecule A[g7]), which have high titers of anti-glucose-6-phosphate isomerase (GPI)-specific autoantibodies, a reduced percentage of Tfh17 cells and upregulation of autoantibody sialylation. Consistent with these findings, an abundance of OX40-overexpressing Tfh17 cells has been observed in RA patients, and their frequency has been negatively correlated with the expression of ST6Gal I [34,35].

### 2.3. The Hyposialylation of Antibodies Results in the Formation of Circulating Immune Complexes and Complement Activation

IgA nephropathy (IgAN) is related to the binding of IgA within the glomerular mesangium, which leads to a diffused glomerulonephritis with mesangial proliferation and a secondary activation cascade of the complement. Abnormalities in IgA1 glycosylation have been described, including hyposialylation. These modifications generate neo-epitopes that are recognized by naturally occurring IgG and IgA1 antibodies, resulting in circulating immune complexes (CIC) and complement activation [36,37]. Moreover, these hyposialylated IgA1 have more avidity for mesangial cells via the transferrin receptor (TfR1), and contribute to IgAN [38].

Primary Sjogren’s syndrome (pSS) is an autoimmune disease characterized by the lymphoid infiltration of exocrine glands. Oligoclonally-activated B cells result in hypergammaglobulinemia, elevated levels of CIC and non-organ specific autoantibodies, such as anti-SS-A/Ro and anti-SS-B/La autoantibodies. The analysis of this hypergammaglobulinemia has demonstrated an abnormally high proportion of asialylated IgG in patients, whereas the IgA1 and IgA2 appear to be hypersialylated. The sera of certain patients simultaneously contain rheumatoid factors, which are antibodies against the Fc portion of IgG. Rheumatoid factors present a strong affinity for asialylated IgG, inducing the formation of CIC. Moreover, a further explanation lies in sialyltransferase activity, which is responsible for excess sialylated IgA in the serum and related CIC in patients. In fact, the asialoglycoprotein receptor (ASGP-R) and the Fc alpha receptor I (FcαRI) bind terminal galactose (Gal) residues of IgA. Oversialylated IgA from patients with pSS masks their terminal Gal residues and is less likely to bind these two receptors compared to native or desialylated IgA, thus preventing the recognition of IgA by the receptors responsible for their clearance, resulting in an excess of serum IgA and related CIC [39,40]. Understanding the mechanisms of gammaglobulin sialylation appears critical to understanding clinical outcomes in patients.

### 2.4. The Hyposialylation of IgG Antibodies Is Responsible for Organ Lesions

SLE is a systemic autoimmune disorder that affects multiple organs and tissues. Autoantibody production and immune complex deposition occur in the target organs, leading to inflammation and damage. Thus, SLE affects the skin, joints, heart, lungs, and brain, with the development of glomerulonephritis in 30–60% of patients. For this disease, a decrease of galactosylation and sialylation of IgG has been described, which contributes to pathophysiological consequences like nephritis [41]. Thereafter, the sialylation of autoreactive IgG antibodies is associated with a reduction in chronic inflammation and nephritis development [42].

### 2.5. The Production of Anti-Sialic Acid Antibodies Leads to the Development of Autoimmune Diseases

Hashimoto’s thyroiditis is an autoimmune disease of the thyroid gland. The *N*-glycolylneuraminic acid (Neu5Gc) is a Sia that is not synthetized by humans. This Sia can be found in red meat or milk products and, consequently, in humans. Neu5Gc-containing molecules trigger an immune system response. Anti-Neu5Gc antibodies can then be produced; they are found in patients with this autoimmune disease and seem to be implicated in its development [43].

Table 1 summarizes the autoimmune diseases in which the modulation of sialylation affects disease activity.

## 3. Sialylation Regulates the Activation of the Immune System

Before mentioning the impact of sialylation on immune cells as well as immunoglobulins (IgG, IgA), a short description of these glycosylation mechanisms follows.

### 3.1. Sialylation Results from a Complex Glycosylation Process

**Figure 1:**
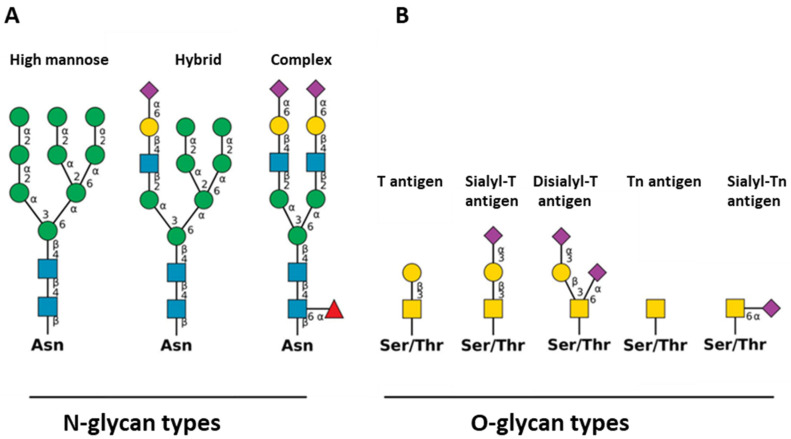
(**A**) *N*-glycan types: high mannose, hybrid, and complex. (**B**) *O*-glycan types: T antigen, sialyl-T antigen, disialyl-T antigen, Tn antigen, and sialyl-Tn antigen. Red triangle for fucose (Fuc); yellow circle for galactose (Gal); blue circle for glucose (Glc); yellow square for *N*-acetylgalactosamine (GalNAc); blue square for *N*-acetylglucosamine (GlcNAc); purple square for *N*-acetylneuraminic acid (Neu5Ac). Asn: asparagine; Ser/Thr: serine/threonine.

Glycoconjugates (i.e., glycoproteins and glycolipids) are found in all living organisms. Among the various protein glycosylation pathways recently reviewed, the most commonly described glycosylation types have been *N*-glycans and mucin-type *O*-glycans (Figure 1) [44].

*N*-glycosylation refers to the co- and post-translational event initiated in the endoplasmic reticulum (ER). In the ER, the stepwise addition of monosaccharides takes place, leading to a preassembled oligosaccharide that is transferred ‘en bloc’ on an asparagine (Asn) residue of the nascent protein by the oligosaccharyltransferase complex. Maturation steps involving the ordered action of glycosyltransferases and glycosidases occur in the Golgi apparatus and give rise to a wide variety of complex oligosaccharides (Figure 1A) [44]. The heterogeneity of *N*-glycans’ synthesis in mammals is dependent on numerous factors, including substrate availability; suitable glycosyltransferases, glycosidases localization and activities; and the transport of glycans from the ER to the Golgi. These structures can be further elongated by the adjunction of Sia to Gal.

The initiation step of mucin-type *O*-glycans’ synthesis in the Golgi involves an *N*-acetylgalactosamine (GalNAc) residue transferred to the hydroxyl group of serine (Ser) or threonine (Thr) residues through the action of peptidyl GalNAc transferases (GALNT1-20) (Figure 1B). The simple GalNAcα1–O-Ser/Thr structure known as the cancer-associated Tn antigen (Figure 1B) can be further elongated with a variety of monosaccharides, including Sia.

**Figure 2:**
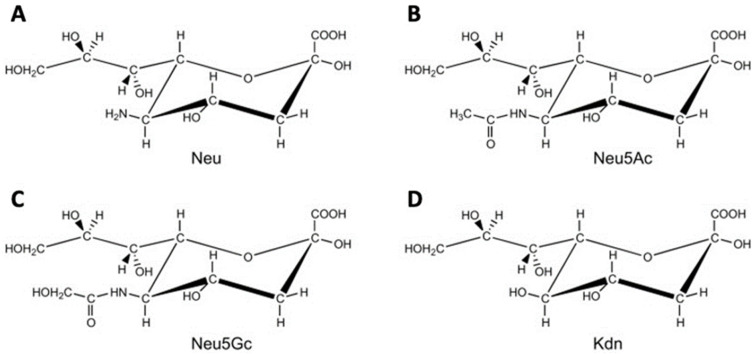
Members of the sialic acid family. (**A**) Neuraminic acid (Neu); (**B**) *N*-acetylneuraminic acid (Neu5Ac); (**C**) *N*-glycolylneuraminic acid (Neu5Gc); (**D**) ketodeoxynonulosonic acid (Kdn).

Sias are monosaccharides with nine carbon atoms that form a large family of more than 40 members. 5-*N*-acetylneuraminic acid (Neu5Ac) is the major Sia found in humans. Neu5Gc and deaminoneuraminic acid (Kdn) are other Sias found in vertebrate species but not or rarely found in humans (Figure 2) [45].

Sialyltransferases (STs) use these monosaccharides as the activated sugar donors, cytidine 5′-monophosphate (CMP)-β-Neu5Ac, CMP-β-Neu5Gc or CMP-β-Kdn for sialylation at the terminal positions of oligosaccharide chains of glycoconjugates [46,47]. The hydroxyl group at position 2 of Sia is most frequently linked to either the 3- or 6-hydroxyl group of Gal residues or the 6-hydroxyl group of GalNAc residues. It can additionally be linked to their 8-hydroxyl group, forming, to a lesser extent, di-, oligo- or poly-Sia chains [46]. STs are classified into four families according to the type of linkage formed and the nature of the sugar acceptor (ST6Gal, ST3Gal, ST6GalNAc and ST8Sia) [48,49] found in the glycosyltransferase family-29 (GT-29) of the Carbohydrate-Active enZYme (CAZy) database [50]. These include six beta-galactoside α2–3-sialyltransferases (ST3Gal I–VI), two beta-galactoside α2–6-sialyltransferases (ST6Gal I–II), six *N*-acetyl galactosaminide α2–6-sialyltransferases (ST6GalNAc I–VI), and six *N*-acetyl neuraminide α2–8-sialyltransferases (ST8Sia I–VI, among which ST8Sia II and ST8Sia IV are polySTs) [51].

### 3.2. Sialylation Modulates Functions of the Immune Cells and Antibodies

#### 3.2.1. Sialylation Modulates Orientation, Homing and the Selection of T Cells

Several studies have shown the importance of sialylation for inhibiting T-cell proliferation and homing; thymocyte selection, tolerance, activation and the orientation of Th1 or Th2 response; and the development of regulatory T cells (T reg) or T cell death [52,53]. For example, in the thymus, ST6Gal I is only detected in mature medullary thymocytes and modifies CD45 *N*-Glycan sialylation. This modification prevents CD45 and galectin-1 linkage, known to induce thymocyte death. This ST is not equally expressed in all thymus compartments and predicts the cell fate easily [54].

Sia interacts with Siglecs, as well, suggesting a potential role in immune modulation. Perdicchio et al. have demonstrated that sialylated antigens are captured by Siglec-E-expressing dendritic cells (DCs). Those types of DCs favor the differentiation of de novo T regs, limiting CD4 and CD8 effector T-cell expansion [55].

Finally, it is interesting to note that sialylation associated with fucosylation plays a further role in T cell activation and Th1/Th2 polarization. In fact, α1,3-fucosyltransferase VII (FucT VII) and ST3Gal IV are absent in naïve T CD4 cells. Their expressions are found during the differentiation of Th1/Th2 and promote the appearance of sialyl-Lewis X (sLe^x^, Neu5Acα2-3Galβ1-4[Fucα1-3]GlcNAc) on Th cells. This molecule allows the binding of Th cells to P-selectin found on the endothelial cell surface and the subsequent migration patterns of Th through an endothelial barrier [56].

#### 3.2.2. Sialylation Inhibits the Activation and Homing of B Cells

ST6Gal I has been found to induce the development and activation of B cells. The former function is mediated by the ST6Gal I extracellular form [57], while the latter function is obtained by modulating CD22 (Siglec-2) molecular activity, a sialic acid-binding lectin that presents at the B-cell surface. The extracellular domain of CD22 specifically recognizes Sia residues and modulates intercellular adhesion as well as the homing and recruitment of B cells. CD22 additionally acts as a negative co-receptor for B cell activation. Sialylation masks CD22 and consequently reduces this function [58]. The additional 6-O-sulfation of Gal leads to α2,6-Sialylated 6′-Sulfo-*N*-acetyllactosamine (found on *O*-glycans), which can additionally modulate the activity of CD22, particularly the homing and recruitment of peripheral B cells [59].

In addition, sialylation affects IgG production by B cell-derived plasmocytes. The IgG sialylated Fc fragment has a diminished affinity for the C1q protein, limiting the activation of the complement and cytotoxicity [60].

#### 3.2.3. Sialylation Induces Phagocytosis and the Homing of Dendritic Cells

Polysialylation (α2,8-linked Neu5Ac chain) is readily detectable at the surface of DCs and is additionally enhanced upon inflammatory stimulation. This polysialylation is observed on CCR7 (C-C chemokine receptor type 7), a receptor for chemokine, and this phenomenon controls DC trafficking by regulating chemokine recognition [61]. Moreover, sialylation modulates the maturation, activation and endocytosis capacity of DCs [62]. In this order, Sia-containing bacteria induce phagocytosis mediated by sialylated receptors on DCs (Siglecs, toll-like receptors), and the presence of sLe^x^ on DCs allows for DC adhesion to endothelium-expressing P-selectins and, consequently, their homing.

#### 3.2.4. Sialylation Modulates Activities of IgG and IgA

##### IgG

**Figure 3:**
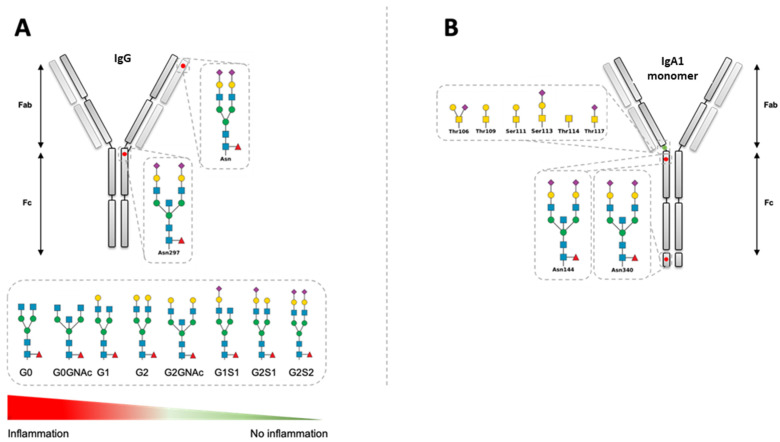
(**A**) IgG glycosylation. (**B**) IgA1 glycosylation. In addition, IgA2 contains two *N*-glycosylation sites, at Asn47 and Asn205. Red triangle for fucose (Fuc); yellow circle for galactose 5Gal); blue circle for glucose (Glc); yellow square for *N*-acetylgalactosamine (GalNAc); blue square for *N*-acetylglucosamine (GlcNAc); purple square for *N*-acetylneuraminic acid (Neu5Ac). Asn: asparagine; Ser/Thr: serine/threonine.

At the Asn 297 position on IgG antibodies, *N*-glycation occurs with an important heterogeneity. More than 30 different variants of IgG glycosylation have been identified. The heptameric core composed of GlcNAc and mannose remains constant. Nevertheless, heterogeneity is brought by adding terminal sugar residues (Sia and Gal) and branching residues (GlcNAc and fucose) [63]. This sugar structure maintains the conformational arrangements of the Fc domains, as well as the hinge regions that participate in the activation of the classical complement pathway and monocyte binding. It has been determined that the presence of terminal Gal or Sia is critical for the launch of pro- or anti-inflammatory reactions [33]. In 15 to 25% of IgG, N-linked glycosylations, which appear during somatic hypermutations, are additionally found in the variable region. They are mainly complex-type biantennary N-linked glycans; Fuc bisecting GlcNAc, Gal, and Sia residues can be found, as well. Il-6 and progesterone, through their receptors on B cells, can modulate glycosylation conformations [64]. The impact of these glycosylations remains inadequately understood. Nevertheless, certain medical conditions help with the understanding of their roles, including how to prevent unwanted reactivity such as during pregnancy (tolerance towards the fetus) and autoimmunity (the lack of Sia on IgG has been associated with the development of RA) [65,66] (Figure 3A).

##### IgA

In a serum, two types of IgA are found in the monomeric form: IgA1 and IgA2. They bind different immune cells, such as neutrophils, eosinophils, monocytes, macrophages and DCs, mainly via the Fc alpha receptor I (FcαRI) [67]. Both IgAs exert opposite immune reactions: while IgA1 is considered an anti-inflammatory antibody, IgA2 is more proinflammatory [68,69]. To explain these observations, the glycosylation of IgA1 and IgA2 was recently studied by Steffen et al. [70]. These two antibodies present two (IgA1) or four conserved sites for *N*-glycosylation (IgA2) [71]. In addition, IgA1 possesses several *O*-glycosylation sites in its hinge region, but Fab glycosylation does not seem to exist on IgA or is highly difficult to detect. Moreover, terminal Sia has been detected to be reduced by half in IgA2 compared to IgA1. In contrast, the signal for free Gal doubles in IgA2 compared to IgA1. In autoimmune diseases, IgA2 are mainly recovered, and the analysis of their glycosylation can provide an explanation. More generally, IgA glycosylation controls IgA effector functions. Figure 3B presents the *O*- and *N*-glycosylation of IgA.

## 4. Increasing Sialylation: A Promising Strategy to Treat Autoimmune Diseases

Currently, injections of intravenous immunoglobulins (IVIgs) are used in autoimmune diseases, and their therapeutic effect is, in part, linked to their rates of sialylation. Based on the observations previously mentioned, other tested therapies are detailed below and in Table 2.

### 4.1. Sialylated Intravenous Immunoglobulins Decrease Inflammation

IVIgs involve the therapeutic preparation of polyclonal IgG obtained from thousands of healthy donors and are successfully used for the treatment of inflammatory and autoimmune diseases at high dosages [72]. Mechanistic studies have revealed that IgG Fc, through FcγRs, modulates the immune response. Once more, these observations have been associated with the sialylation of the IgG. It is interesting to note that the Fc fraction is sialylated and IgGs induce a Th2 response. IL-33 is generally associated with Th2 cytokines, including IL-4, which upregulates FcγRIIB (receptor inhibitor of cell activation) on effector macrophages, contributing to suppressing inflammation [73,74,75]. These observations have been confirmed in Guillain-Barré syndrome. Different fractions of total IVIg, sialylated IVIg and unsialylated IVIg have been tested in an antibody-mediated nerve injury mouse model. The unsialylated fractions of IVIg have not beneficial, while tenfold lower doses of sialylated IVIg compared to whole IVIg have provided an equivalent efficiency [76]. The development of this therapy has been optimized by Washburn et al. with the use of a controlled tetra-Fc sialylation of IVIg-enhancing anti-inflammatory activity, up to 10-fold, across different animal models [77].

### 4.2. Sialylation of Autoantibodies Decreases Their Proinflammatory Activities

Oefner et al. have developed a murine autoimmune model with chicken ovalbumin (OVA). Under inflammatory conditions with lipopolysaccharide or Freund’s adjuvant, plasma cells express low levels of ST6Gal I and produce desialylated IgGs. In contrast, plasma cells induced in tolerogenic conditions have a normal expression of ST6Gal 1 and secrete sialylated IgGs that inhibit antigen-specific T- and B-cell responses [78]. These observations have defined several potential new strategies for treating autoimmune diseases. The injection of artificially-generated sialylated IgG autoantibodies leads to a reduced expansion of Th1, Th17 and B-cell response in mouse models of lupus nephritis and RA [42]. Another strategy has been developed by Pagan et al., who have used a novel construction based on the fusion of the soluble domain of ST6Gal I and the Fc of an IgG1 in another autoimmune mouse model. Engineered solubilized glycosyltransferases have been used to add Sia to IgG in order to convert IgG hyposialylated into anti-inflammatory mediators. To have a sufficient amount of bound Sia, a construction allowing the galactosylation of proteins can be added and the binding of Sia achieved from a terminal galactose. With these two constructions, a significant reduction of inflammation has been observed to a level similar to the one obtained with IVIg [79].

### 4.3. Sialic-Acid-Modified Antigens Allow for the Induction of a Tolerogenic Environment

Knowing that sialylation alters immune responses and autoimmunity, Perdicchio et al. have used sialylated OVA in a mouse model of autoimmunity. Sialylated OVA, via Siglec-E, induces tolerogenic DCs, the generation of T reg cells and the inhibition of new effector IFNγ-producing (interferon-gamma) Th1 cells. Moreover, priming DCs with the Sia-antigen significantly reduces the expansion of myelin-reactive T cells found in experimental autoimmune encephalomyelitis mouse models. These are promising new approaches to treat autoimmune diseases and allergies [55].

### 4.4. The Modulation of Lectins Reduces the Activation of Inflammatory Cells

#### 4.4.1. CD22 Linked to Sialic Acid Induces the Inactivation of B Cells

IVIgs have been demonstrated to modulate BCR signaling through CD22 and promote the apoptosis of mature B cells. The presence of Sia on IVIgs recognized by CD22 has been suggested to explain this phenomenon [80]. The modulation of CD22 in mouse models by an α2,6-sialylated glycan or an anti-CD22 antibody conjugated to an immunotoxin has been tested to eliminate B cells in cases of acute lymphoblastic leukemia. A significant decrease in leukemia cells in bone marrow has been observed [81].

#### 4.4.2. Sialic Acid Micelles Enhance Anti-Inflammatory Efficacy by Their Ligation to E-Selectin

Using an RA OVA mouse model, micelles containing Sia and dexamethasone (DXM) have been used to treat mice. The Sia delivery to E-selectin receptors on inflamed cells reduces inflammation and induces bone repair activities [82]. In this model, another type of micelle (DXM palmitate-loaded liposomes decorated with Sia) has been tested and improved, as well, to reduce RA activity [83]. The same type of experimentation has been conducted in a mouse model of acute kidney injury. Sia-polyethylene glycol-DXM micelles or Sia-modified solid lipid nanoparticles that target E-selectin have been used and, once more, anti-inflammatory effects have been obtained [84].

### 4.5. Adjunction of Oestrogen Stimulates the Sialylation of Antibodies

Using the RA OVA mouse model, postmenopausal mice have been treated with estrogen. Estrogen increases the expression of ST6Gal I in plasmablasts, and that is responsible for adding Sia residues to IgG. The authors observed a significant regression of RA [85].

## 5. Conclusions

Sialylation clearly plays a critical role in autoimmune diseases, and controlling it may become a new interesting therapeutic strategy, along with the use of soluble ST. An improved understanding of the physiological role played by other types of monosaccharides (mannose, fucose, galactose, and so on) and their impact on the modulation of the immune response is critical for the development of new potential treatments. Determining the glycophenotypes of patients’ cells or carrying out the glycosylation mapping of patients’ antibodies could provide for personalized approaches to treating specific patients. Recently, it has been demonstrated that sialylation influences the efficiency of anti-CD20 (rituximab) therapeutic treatments in chronic lymphocytic leukemia, suggesting that targeting glycosylation in cancer pathologies could be another interesting approach [86].

## Figures and Tables

**Table 1:** Importance of sialic acid modifications in autoimmune diseases and pathological consequences.

Disease	Type of Sialylation	Consequences of Sialylation Modification	References
Chronic inflammatory demyelinating polyneuropathy	Hyposialylation of IgG Fc	Correlated with the severity of the disease	[24]
Granulomatosis	Hyposialylation of anti-proteinase 3 antibodies	Correlated with the severity of the disease Enhanced inflammatory environment	[25]
Rheumatoid arthritis	Hyposialylation of anti-citrullinated protein antibodies	Enhanced inflammatory environment	[31]
IgA nephropathy	Hyposialylation of autoantigen-reactive IgA antibodies	Formation of immune complexes and renal toxicity by complement activation	[36,37]
Primary Sjogren’s syndrome	Asialylated IgG IgA1 and IgA2 are oversialylated	Circulating immune complexes composed of asialylated IgG and rheumatoid factors Excess of IgA and circulating immune complexes	[39,40]
Systemic lupus erythematosus	Hyposialylation of autoantigen-reactive IgG antibodies	Nephritis	[41]
Hashimoto’s disease	*N*-glycolylneuraminic acid	Excess of anti-*N*-glycolylneuraminic acid Development of the disease	[43]

**Table 2:** Therapies developed in autoimmune diseases.

Type of Therapies	Therapy’s Consequences	References
Sialylated intravenous immunoglobulins	Modulation of innate immune effectors Induction of Th2 response with suppression of inflammation	[72,73,74,75,76,77]
Sialylation of pathogenic antibodies	Lower frequencies of pathogenic Th1, Th17 and B-cell responses Reduction of inflammation	[42,78,79]
Sialic-acid-modified antigens	Induce tolerogenic dendritic cells Produce T reg cells Inhibition of new effector T cells and of Th1 IFN-γ production	[55]
Modulation of CD22	Induce inactivation of B cells	[80,81]
Sialic acid micelles/binding synthetic peptide	Induce anti-inflammatory activities	[82,83,84]
Estrogen treatment	Enhance the sialylation of antibodies	[85]

## Data Availability

Not applicable.
